# The Femoral Neck Mechanoresponse to Hip Extensors Exercise: A Case Study

**DOI:** 10.1155/2017/5219541

**Published:** 2017-01-11

**Authors:** Saulo Martelli, Hossein Mokhtarzadeh, Peter Pivonka, Peter R. Ebeling

**Affiliations:** ^1^School of Computer Science, Engineering and Mathematics, Flinders University, Adelaide, SA, Australia; ^2^North West Academic Centre, The University of Melbourne, St Albans, VIC, Australia; ^3^Department of Orthopedic Surgery, Harvard Medical School and Center for Advanced Orthopaedic Studies, Beth Israel Deaconess Medical Center, Boston, MA, USA; ^4^Department of Mechanical Engineering, Melbourne School of Engineering, University of Melbourne, Parkville, VIC, Australia; ^5^Australian Institute for Musculoskeletal Science, St Albans, VIC, Australia; ^6^St Vincent's Department of Surgery, The University of Melbourne, Fitzroy, VIC, Australia; ^7^Department of Medicine, School of Clinical Sciences, Faculty of Medicine, Nursing and Health Sciences, Monash University, Clayton, VIC, Australia

## Abstract

Physical activity is recommended to prevent age-related bone loss. However, the proximal femur mechanoresponse is variable, possibly because of a muscle-dependant mechanoresponse. We compared the proximal femur response with the femoral strain pattern generated by the hip extensor muscles. A healthy participant underwent a six-month unilateral training of the hip extensor muscles using a resistance weight regularly adjusted to the 80% of the one-repetition maximum weight. DXA-based measurements of the areal Bone Mineral Density (aBMD) in the exercise leg were adjusted for changes in the control leg. The biomechanical stimulus for bone adaptation (BS) was calculated using published models of the musculoskeletal system and the average hip extension moment in elderly participants. Volumetric (ΔvBMD) and areal (ΔaBMD) BMD changes were calculated. The measured and calculated BMD changes consistently showed a positive and negative effect of exercise in the femoral neck (ΔaBMD = +0.7%; ΔvBMD = +0.8%) and the trochanter region (ΔaBMD = −4.1%; ΔvBMD = −0.5%), respectively. The 17% of the femoral neck exceeded the 75th percentile of the spatially heterogeneous BS distribution. Hip extensor exercises may be beneficial in the proximal femoral neck but not in the trochanteric region. DXA-based measurements may not capture significant aBMD local changes.

## 1. Introduction

Muscle forces contribute to maintaining the bone mass by modulating bone adaptation [1]. Physical activity has often been prescribed to prevent the age-related bone loss [2] and the associated increase of the risk of fracture [3]. The proximal femur is one of the most clinically relevant anatomical sites due to its high rate of bone loss [3] and the high number of fracture events and the severity of fracture [4]. However, different exercise interventions yielded variable femoral neck mechanoresponse to activity, ranging from no response to a significant increase in bone mass [2, 5–7]. To date, no study compared the proximal femur response in elderly women and the femoral mechanics for a specific activity.

The femoral neck is characterised by a variable cortical thickness reaching its thinnest region in the proximal neck where fracture is most likely to occur [8, 9]. Several studies investigated the response of the proximal femur to different types of exercise showing variable results [5–7]. For example, changes of the areal Bone Mineral Density (aBMD) following a 6-month unilateral hop exercise intervention were measured using DXA [7] in healthy premenopausal women; the femoral neck aBMD increased on average from 0% to 1.8% across participants exercising two and seven days per week while intraparticipant aBMD changes ranged from −3.4% to +3.8% in the trochanter and the upper neck region, respectively [7]. In postmenopausal women, 11-month, three-sessions-per-week, 45-minutes-per-session exercise including walking, jogging, and stairs induced a 3.5 ± 0.8% and a 6.1 ± 1.5% aBMD increase in the femoral neck and Ward's triangle whereas weight-lifting and rowing induced a 5.1 ± 2.1% aBMD increase in Ward's triangle but no aBMD changes in the femoral neck and the trochanter [6]. Computed-tomography was used to measure the volumetric BMD (vBMD) changes following a 16-week, three-sessions-per-week, 45-minutes-per-session exercise interventions in two cohorts of mixed males and females (25–55 years of age). One cohort executed abduction/adduction exercises showing an increase of the cortical volume in the trochanter whereas the second cohort executed squat/deadlifts exercises showing an increase of the cortical volume in the femoral neck and a concomitant cross section area and lean-tissue fraction increase in the hip extensor muscles [5]. Based on these findings it appears that the proximal femur adaptation response is determined by concurring factors such as age [4] and diet [6] and genotype [7] and exercise intensity [10] and frequency [7] and that the hip extensor muscles have the potential to generate a muscle-specific and spatially heterogeneous mechanical stimulus for bone adaptation in the proximal femur [5]. However, to the best of our knowledge, no study has investigated the association between the mechanical stimulus for bone adaptation and bone response induced by maximal contractions of the hip extensor muscles in the proximal femur. We hypothesise that contractions of the hip extensor muscles may induce a beneficial bone response in a very critical region (i.e., the proximal neck) and that the average DXA-based aBMD measurement in the femoral neck and trochanteric and intertrochanteric region may not capture a localised mechanoresponse arising from a heterogeneous distribution of the bone mechanical stimulus for bone adaptation [5].

It has been inherently difficult to quantify the bone response and the mechanical stimulus in the proximal femur generated by specific muscle groups, mostly because of (1) the time required for bone adaptation to take place (i.e., ≥6 months) [6, 7], (2) a generally moderate bone response of comparable magnitude of the accuracy of DXA devices [11], (3) the fact that the different muscles normally active during a generic activity may induce different, spatially heterogeneous, mechanical stimuli for bone adaptation [5], and (4) the fact that it is difficult to measure bone strain in vivo [12]. As a first step, the pattern of the mechanical stimulus and bone response can by studied by combining a longitudinal exercise intervention and computational models in selected individuals. A six-month longitudinal study based on three sessions per week of intense unilateral exercises of a healthy premenopausal woman can be used to induce bone changes in the proximal femur [7]. A leg-press training protocol can be designed to target the hip extensor muscles. The aBMD in the femoral neck and trochanteric and intertrochanteric regions can be measured using DXA at training commencement and completion in both the exercise and control leg. Changes of aBMD in the exercise leg can be calculated and adjusted for aBMD changes in the control leg to minimise confounding effects such as that of age or diet [7]. The force of the hip extensor muscles exerted during maximal hip extension exercises [13] can be calculated using musculoskeletal modelling. The mechanical stimulus for bone adaptation and the vBMD response in the femoral neck and trochanteric and intertrochanteric regions can be calculated using a finite-element model and bone adaptation theory [1, 12, 14] and matched to the measured aBMD for the following: (a) comparing the pattern of the measured and calculated bone response and (b) analysing the distribution of the mechanical stimulus.

The aim of the current study was to compare the bone response in the proximal femur to intense contractions of the hip extensor muscles with the deformation pattern generated by the same muscle group. The femoral response to hip extensor exercises was measured using common DXA procedures and compared to the mechanical stimulus calculated using bone adaptation theory.

## 2. Methods

A healthy premenopausal Caucasian woman of average weight and height (40 years of age, 161 cm height, and 67.6 kg weight) underwent a longitudinal unilateral training of the hip extensor muscles informed by the work of Bailey and Brooke-Wavell [7]. Training was conducted at the gym located in the Parkville Campus of The University of Melbourne (Parkville, VIC, Australia). DXA images were taken at the NorthWest Academic Centre, Melbourne Medical School, Faculty of Medicine, Dentistry, and Health Sciences, The University of Melbourne (Sunshine Hospital, St. Alban, VIC, Australia). Ethics approval for the study was obtained from the Melbourne Health Human Research Ethics Committee. The participant gave informed consent prior to participating in the study.

Recruitment of the participant was conducted using the University of Melbourne staff newsletter. Eligibility criteria were (a) ability to perform intense impact activity; (b) no reported medical problems potentially altering bone metabolism, lower limb, or back pain problems during the previous 12 months; (c) adequate calcium from dairy products; (d) regular menstrual cycles; (e) not having been pregnant nor having given birth or lactated; (f) sedentary lifestyle during the same previous 12 months, meaning no participation in high-impact or weight-bearing exercises for more than one hour per week. The participant was matched to the average weight and height of Caucasian women in Australia (Australian Bureau of Statistics; http://www.abs.gov.au/).

**Figure 1:**
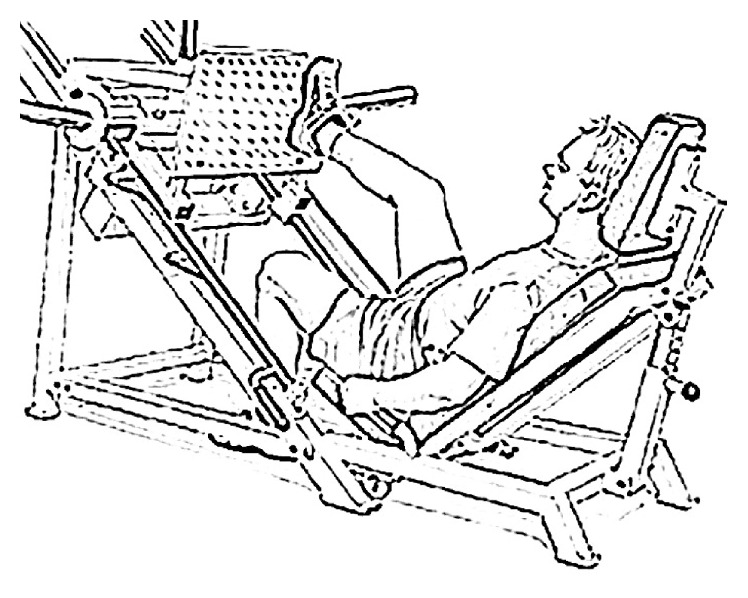
A sketch representation of the unilateral exercise type targeting the hip extensor muscles investigated in the present study.

The exercise intervention was a unilateral longitudinal trial involving a six-month training targeting the hip extensor muscles for no less than four sessions per week. The participant was instructed in the correct execution of the exercise and supervised by the gym staff over the entire training. Each training session involved a gentle warm-up and mobilisation exercises followed by 5 sets of 10 unilateral exercises on a leg-press machine (Linear Leg Press, Hammer Strength, Life Fitness, Rosemont, USA) available at the gym (Melbourne University Sport, Parkville, VIC, Australia). The participant chose the leg to be exercised for the entire study duration and was instructed to sit comfortably in the machine, to use a medium foot position, to place feet as high as possible on the foot plate, to push through the heels, and to use full range of motion from nearly reaching the chest with the knees to full leg extension (Figure 1). The participant was also instructed to comfortably and safely place the nonexercise limb out of reach of the leg press mechanism and to stop exercising in case of pain. For the first two weeks, each session was composed of 2 sets of 10 unilateral submaximal exercises. One set of exercises per session was added for the third, fourth, and fifth week. Five sets per session were executed for the rest of the study. Each training session was composed of an initial warm-up and relaxation and stretching exercises between sets and at end of session for a total of approximately 90 minutes. The weight was set to 80% of the weight lifted during a one-repetition maximum (1-RM) exercise, defined as the heaviest weight one can lift once, updated fortnightly for the first two months and monthly for the remaining of the training. To reduce the risk of injury in the execution of 1-RM exercises, the 1-RM weight was estimated using a published relationship taking as input a submaximal weight and the maximal number of exercises the volunteer could execute [15]. For the first test, the participant chose a weight she could comfortably lift for more than ten times, whereas for the subsequent 1-RM tests the weight was set to be equal to that used during the previous session. The amount of exercise, the weight lifted, and a subjective rating of the exercise difficulty, a number form zero (easy) to ten (difficult), was recorded in training logs. The participant was requested to avoid strenuous exercise, alcohol, and caffeine intake during the preceding 12 h, to provide individual training logs, and not to commence an exercise program involving long-distance running, jogging, or other activities involving intense exercises or impact. Complementary activities involving light symmetric activities like walking, cycling, swimming, or pilates were recommended to maintain a proper body symmetry and balance. A home-based strengthening exercise was recommended to speed up recovery of residual strength asymmetries between legs at study completion. The aBMD in the femoral neck and trochanteric and intertrochanteric region at both hips was measured using two DXA (QDR 4500, Discovery; Hologic Inc., Bedford, Massachusetts, USA) imaging sessions, both conducted by professional staff following the Hologic guidelines. The first DXA was taken at training commencement (*t*_1_ = 1 day) and the second DXA was taken at training completion (*t*_2_ = 6 months). The DXA machine was routinely recalibrated at six-month intervals, therefore minimizing the eventual long-term drift of DXA measurements [16].

Compliance was determined by calculating the percentage ratio between the amount of exercise executed and the total amount in the training protocol. The Body Mass Index at training commencement and completion was calculated using the body weight (kg) and height (m) listed in the DXA reports. The aBMD changes in the femoral neck and trochanteric and intertrochanteric regions were extracted from the DXA reports taken at training commencement and completion. The aBMD changes in the exercise leg were adjusted for changes in the control leg [7, 17]. The measured aBMD changes were compared to the short-term coefficient of variance (CV) of DXA measurements in our laboratory, which was 1.5% [18].

The computational model utilized in our study included a lower-limb musculoskeletal model and a finite-element model of the right femur from an average sized female donor (167 cm height; 63 kg weight) with no reported history of musculoskeletal diseases prior to death [12, 14]. The femoral length, head diameter, femoral neck length, craniocaudal angle, and anteversion angle were within 1.2 standard deviation from the average 60-year-old Caucasian woman [19–21] while the aBMD in the femoral neck was 0.53 g·cm^−2^, one standard deviation below the average 85-year-old Caucasian woman. In summary, the musculoskeletal model was a 12-segment, 18-degrees-of-freedom articulated system, actuated by 82 Hill-type muscle-tendon units. The hip and the knee were modeled using ideal ball-and-socket and hinge joints. The hip center was defined as the center of the sphere that best fits the femoral head surface. The knee axis was assumed to pass through the femoral epicondyles. The hip extensor muscles comprised the gluteus maximus, semimembranosus, semitendinosus, and biceps femoris long head. The gluteus maximus was modelled using three separated lines of action representing the anterior, intermediate, and posterior fibre bundles while the semimembranosus, semitendinosus, and the biceps femoris long head were modeled using a single line of action. The attachment points were defined as the mean point of the digitized attachment area during dissection and the muscle path was defined using the digitized superficial muscle fiber path as reference [22]. The finite-element model was based on the computed-tomography (CT) of the donor's right femur. Bone tissue material properties were assigned to each (10-node tetrahedral) element of the unstructured finite element mesh. The (element-by-element) homogenous isotropic Young modulus was derived from the CT grey levels using a validated procedure [23]. Correlation analysis of calculated and measured cortical bone strains showed an average error of 84 *με* and a slope of 1.15 [12]. Patterns of the hip reaction force were in good agreement with public measurements for five different types of activities recorded on patients who received a total hip replacement [12].

**Figure 2:**
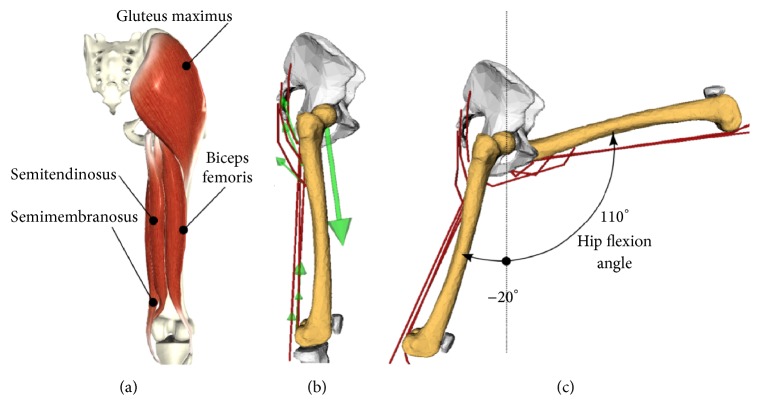
From the left hand side, (a) the anatomical representation (posterior view) of the hip extensor muscles taken from BioDigital Human (https://human.biodigital.com); (b) the model in the anatomical position (lateral view) including the muscle and hip forces (in green); and (c) the range of hip flexion angles investigated.

The peak muscle force of the hip extensor muscles was uniformly scaled to match, at full activation, the average quasi-static isokinetic peak hip extension torque (105.1 Nm) measured on a cohort of healthy elderly controls (54 to 73 years of age) reported by Steinhilber et al. (2011) [13]. The isolated effect of maximal isometric hip extensor contraction was calculated assuming no passive muscle resistance to muscle stretching and zero activation of the antagonist muscles and by neglecting gravitational forces. The range of the hip flexion angle (i.e., −20°–100° hip flexion angle) was explored using fifteen intermediate angles (Figure 2). All the other joint angles were set to zero. The hip extensor muscles were set fully activated and the hip contact force was calculated by solving for static equilibrium of the femur. Muscle and joint forces were applied to the finite-element model using an in-house subroutine [12].

Finite-element simulations were run using the direct solver implemented in Abaqus© (Dassault Systèmes, USA). The biomechanical stimulus (BS) for bone adaption was calculated according to Frost's mechanostat theory [1], which states that bone adapts to changes in the mechanical environment in order to maintain a homeostatic bone mineral density pattern. Strain energy was used to represent the mechanical stimulus for bone adaptation according to the work of Huiskes et al. (1987) [24]. Walking was assumed to generate the homeostatic strain energy (SE_W_) pattern in sedentary volunteers [12]. The biomechanical stimulus (BS) was

(1) $$\begin{array}{c} BS=\frac{\left(SE_{HE}-SE_{W}\right)}{SE_{W}}, \end{array}$$

where SE_HE_ is the element-by-element strain energy induced by the hip extensor (HE) muscles averaged over the studied hip flexion angles and SE_W_ is the element-by-element strain energy averaged over the stance phase of walking taken from our previous work [12]. The proximal femur finite-element model was partitioned into the femoral neck and trochanteric and intertrochanteric region in order to compare the obtained in silico adaptation responses with the respective in vivo aBMD measurements in the DXA report. The change in volumetric Bone Mineral Density (vBMD) was calculated as

(2) $$\begin{array}{c} {\Delta vBMD}_{j}=k\times \overset{}{\underset{V_{j}}{\int }}BS\times dV_{j}, \end{array}$$

where ΔvBMD is the change in vBMD, the subscript *j* represents the specific region of interest (i.e., the femoral neck and trochanteric and intertrochanteric region); *V*_*j*_ is the volume of the *j*th region of interest,* k* is a constant scaling factor representing the bone mechanosensitivity in the respective region, that is, the amount of bone that is deposited or resorbed based on a positive ((SE_HE_ − SE_W_) > 0) or negative ((SE_HE_ − SE_W_) < 0) biomechanical stimulus; *k* was assumed to be the same in all three regions and calculated based on the assumption that vBMD changes were equal to the adjusted aBMD changes in the intertrochanteric region where small to negligible BMD changes are expected [5]. The factor *k* was then used to calculate vBMD changes in the femoral neck and trochanteric region using (2).

The participant's height and weight were compared with the distribution of Australian women (Australian Bureau of Statistics; http://www.abs.gov.au). The participant compliance, Body Mass Index (BMI), the average subjective indicator of the exercise difficulty, and the adjusted aBMD changes in the femoral neck, trochanteric, and intertrochanteric region were calculated. The measured aBMD changes were compared with the short-term CV of the DXA machine. The magnitude of the hip contact force, the peak tensile and compressive strain, strain energy density, and von Mises stress over physiological hip flexion angles were plotted against the studied hip flexion angles. The spatial distribution of the calculated biomechanical stimulus for bone adaptation was plotted and the volume fraction exceeding the 75th percentile of the biomechanical stimulus distribution in the femoral neck, trochanter, and intertrochanteric region was calculated. The calculated vBMD changes were compared with the adjusted aBMD changes (Table 1).

## 3. Results

### 3.1. In Vivo Study

**Figure 3:**
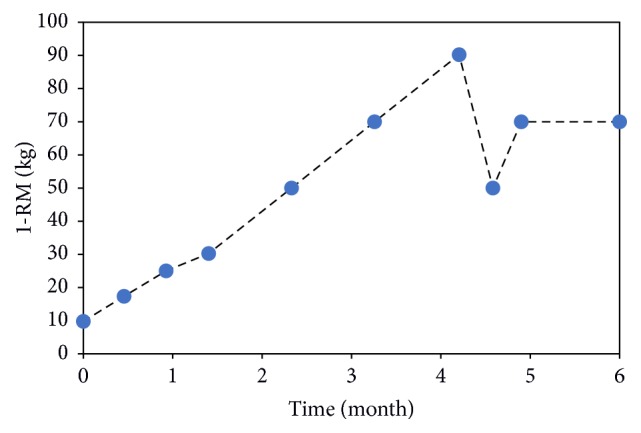
The weight lifted over the course of training. Following the two-week interruption of training, the weight was set to 50 kg (time = 4.75 months) and to 70 kg during the last month training.

The selected participant was a healthy woman (161 cm height; 67.6 kg weight) who matched the average height and weight in the Australian population (161.4–162.1 cm height; 67.0–70.1 kg weight; Australian Bureau of Statistics: http://www.abs.gov.au). No signs of anatomical or bone density abnormalities were evident from the DXA report. Participant compliance was 97%. The resistance weight increased from 10 kg to 90 kg after nineteen weeks of training, when a two-week interruption was necessary due to the insurgence of back-pain for unrelated causes. For safety reasons, training was then completed using a reduced 70 kg resistance weight (Figure 3). The average subjective indicator of the exercise difficulty was 8.5. The participant BMI decreased from 26.0 to 24.9 kg×m^−2^. On average, aBMD in the exercise hip decreased by −2.1% whereas a −1.2% aBMD decrease was found in the control hip (Table 1). Adjusted aBMD changes (ΔaBMD) were site-specific showing a relative 0.7% aBMD increase in the femoral neck, a 4.1% aBMD decrease in the trochanteric region, and a 0.1% increase in the intertrochanteric region (Table 1). These values were higher compared to the CV in the trochanteric region, but not in the femoral neck and in the intertrochanteric region.

### 3.2. In Silico Study

**Figure 4:**
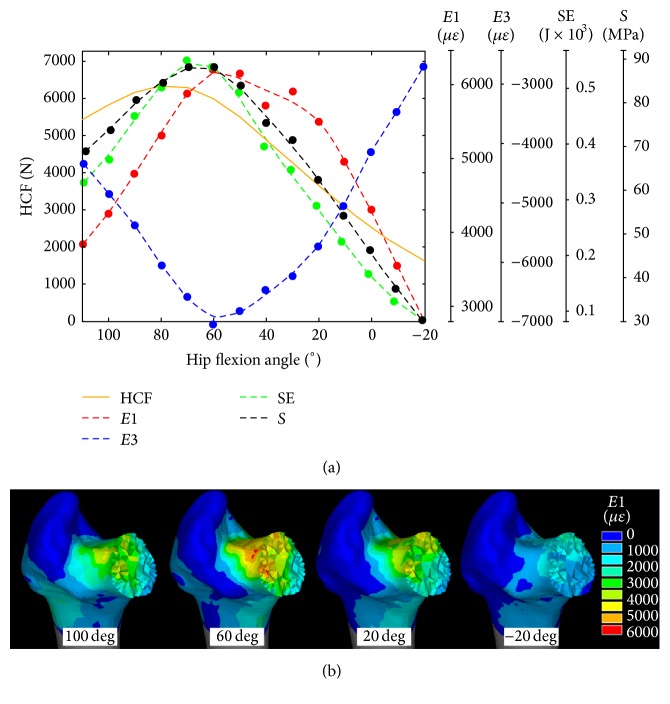
(a) The magnitude of the hip contact force (HCF), the peak tensile strain (*E*1), compressive strain (*E*3), strain energy (SE), and von Mises stress (*S*) over the studied range of hip flexion angles. (b) The tensile strain distribution in the proximal femur.

**Figure 5:**
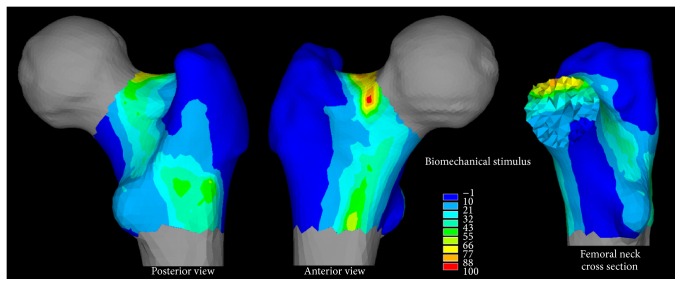
The biomechanical stimulus for bone adaptation. From the left hand side, a frontal view, a posterior view, and the distribution of the biomechanical stimulus over a medial cross section of the femoral neck region.

The peak of the hip contact force was 6340 N calculated with the hip 80 degrees of flexion. The femoral neck was subjected to tensile and compressive loads in the proximal and distal neck and the intertrochanteric region was medially in compression and laterally in tension while low loads were found in the trochanteric region. The principal tensile strain was located in the proximal neck throughout the studied hip flexion angles, reaching 6104 *με* at 60 degrees of hip flexion (Figure 4). The biomechanical stimulus (BS) for bone adaptation reached values between 80 and 100 across the proximal femoral neck. Moderate negative values of the biomechanical stimulus, indicating the potential for bone resorption, were found in the trochanteric region, the medial neck, and the medial intertrochanteric region (Figure 5). Patterns of calculated vBMD changes compared well with patterns of the adjusted aBMD changes, both showing an increase in the femoral neck (ΔaBMD = +0.7%; ΔvBMD = +0.8%) and a decrease in the trochanteric region (ΔaBMD = −4.1%; ΔvBMD = −0.5%). The 17%, 32%, and 22% of the femoral neck and trochanter and intertrochanteric regions exceeded the 75th of the distribution of the biomechanical stimulus for bone adaptation.

## 4. Discussion

We studied the mechanical effect of hip extensors contraction in the proximal femur for an average anatomy and peak loads in an adult woman. Bone changes in the femoral neck and trochanteric and intertrochanteric regions were measured using DXA before and after training of the hip extensor muscles while the biomechanical stimulus for bone adaptation and the related vBMD changes were calculated using bone adaptation theory [1]. Experimental and theoretical results consistently showed spatially heterogeneous pattern of bone changes over the proximal femur causing a BMD increase in the femoral neck and a BMD decrease in the trochanter. The calculated distribution of the biomechanical stimulus for bone adaptation suggests that common integral DXA measurement of the femoral neck aBMD may not capture a localized, and yet important, stimulus for bone apposition in the very proximal neck.

Experimental and theoretical analyses consistently showed a pattern of BMD changes over the proximal femur characterised by a positive effect of the exercise intervention in the femoral neck (ΔaBMD = +0.7%; ΔvBMD = +0.8%) and a BMD decrease in the trochanter (ΔaBMD = −4.1%; ΔvBMD = −0.5%). Although the moderate aBMD increase measured in the femoral neck may not be biological significant because it is smaller than the short-term CV (1.5%) of the utilized DXA device [18], the relative aBMD changes between the femoral regions (i.e., femoral neck, trochanter, and intertrochanter) were biologically significant providing confidence on the measured regional pattern. This pattern is consistent with the positive effect of the hip extensor muscles in the femoral neck, but not in the trochanter, according to the work by Lang et al. (2014) [5] who used CT imaging as the end point for assessment. Therefore, exercising the hip extensor muscles may help mitigating the age-related increase of the risk of femoral neck fractures whereas other exercise types such as hip abduction exercise may help mitigating the risk for injury in the trochanteric region. The calculated bone response over the femoral neck volume was spatially variable showing a high mechanical stimulus for bone apposition localized in the very proximal neck (Figure 5). Therefore, the most commonly used DXA-based aBMD measurements may not be capable of capturing a localized and yet important bone response by providing integral values calculated over large bone portions [5]. Computed-tomography is a viable solution to study the spatially heterogeneous bone response to exercise in the proximal femur [5]. Another positive effect of training was the ninefold increase of the resistance weight, which may imply increased muscle strength and mobility that may help reducing the propensity to fall and the related risk of fracture [25]. However, the ninefold increase of the resistance weight may not only reflect a muscle strength increase but also contain the effect of an increased confidence in executing maximal exercises. Finally, the proximal femoral neck was loaded in tension across the studied hip flexion angles reaching the peak load in vicinity of the peak of the hip contact force (Figure 4), therefore in vicinity of the participant's maximal effort. This information can be used in the design of exercise training protocols targeting the hip extensor muscles.

To the best of the authors' knowledge this is the first study combining DXA measurements of bone response in the proximal femur with computer models for describing the distribution of the biomechanical stimulus during isolated contraction of the hip extensor muscles. The present findings confirm the capability of hip extensor muscles to induce high localized loads in the femoral neck [12] and provide information about the distribution of the biomechanical stimulus for bone adaptation generated by the hip extensor muscles in the whole proximal femur throughout a physiological range of hip flexion angles. The positive effect of hip extensor contractions confirmed the notion by Kelley et al. (2001) [26] that resistance exercises can promote femoral strength. The adjusted ΔaBMD in the femoral neck (+0.7%) and in the trochanter (−4.1%) are in agreement with the average effect in the femoral neck (ΔaBMD = +0.9%) and the lowest intraparticipant effect in the trochanter (ΔaBMD = −3.4%) reported by Bailey and Brooke-Wavell (2010) [7] using the same longitudinal study design used here. Lastly, the good agreement between the pattern of the calculated biomechanical stimulus and the measured aBMD changes strengthens the notion, introduced by Frost (1987) [1], that muscles, loads, and bone changes are correlated.

There are several limitations to the present study. Firstly, the present study was based on a single subject limiting the generality of the conclusions. More research is necessary to understand the interaction between anatomical and physiological factors in determining the bone response to specific loading scenarios across individuals. Secondly, the exercise was conducted by a 40-year-old healthy woman while older postmenopausal woman, most at risk for hip fractures, may experience a far slower bone adaptation response. While exercising earlier in life can prevent excessive bone loss later in life, the optimal age-dependent training length needs to be determined. Thirdly, exercising did not prevent bone loss as appears evident from the reported absolute aBMD changes (Table 1), thus raising concerns about the actual efficacy of the specific exercise type we investigated. More prolonged training and drug and food supplements may lead to higher osteogenic activity, possibly reversing bone loss [6, 27]. Fourthly, the maximal hip extension exercise may have involved activity of the antagonistic muscles possibly altering the mechanical stimulus generated by the agonist muscles. However, the contribution to the net joint moment of the antagonist muscles is expected to be less than the 20% of the corresponding contribution of the agonist muscles [28]. Fifthly, the pixel-by-pixel aBMD value from the DXA images was not available complicating a more in-depth comparison of the local aBMD changes with a patient-specific estimation of bone mechanics. However, the model used in the present study was shown capable of predicting realistic patterns of femoral strain during activity [12, 14] allowing the comparison of the observed bone changes with generic patterns of bone mechanics, rather than their absolute values. This is important because of the spatially heterogeneous and exercise type dependent bone response in the proximal femur [5]. Sixthly, different bone remodelling algorithms have been proposed in the literature [29–32]. However, the algorithm used in the present study has been shown to provide a realistic representation of bone changes in humans [29]. Finally, aging induces bone loss particularly localized in the inner trabecular network and a concomitant increase of the periosteal diameter [3, 4] likely altering bone mechanics. More research is necessary to understand the effect of age-related bone changes on the mechanical stimulus for bone adaptation.

Given the above limitations, the current results provide a general understanding of the mechanical effect of the hip extensor muscles in the proximal femur. The present study provides a theoretical foundation supporting the observed bone response in the proximal femur during training of the hip extensor muscle [5] and it provides a quantitative explanation for the variable and generally weak femoral neck response observed using common DXA methods. More work is necessary to quantify the mechanoresponse in the proximal femur on a participant-by-participant base. Another possible impact of this study is in its showing the use of modelling techniques for investigating the site- and muscle-specific contribution to bone biomechanics, which can support the design of future exercise interventions targeting bone changes in specific anatomical regions.

In conclusion, training of the hip extensor muscles induces spatially heterogeneous bone changes in the proximal femur and may help contrasting the age-related bone loss in the very proximal neck, a region of critical importance to hip fragility fractures [8, 9]. Standard DXA examinations may not provide an accurate representation of highly localized femoral neck changes caused by the highly localized mechanical stimulus generated by the hip extensor muscles.

## Figures and Tables

**Table 1:** Absolute and adjusted bone changes in the proximal femur. Values represent the percentage change of area, Bone Mineral Content (BMC) and areal Bone Mineral Density (aBMD) at six-month training (*t*_2_) with respect to the same values measured at training commencement (*t*_1_).

	Absolute bone changes (*t*_2_-*t*_1_)						Adjusted bone changes (*t*_2_-*t*_1_)		
	Exercise leg			Control leg					
	Area (cm^2^)	BMC (g)	aBMD (g/cm^2^)	Area (cm^2^)	BMC (g)	aBMD (g/cm^2^)	Area (cm^2^)	BMC (g)	aBMD (g/cm^2^)
Neck	−1.60%	−3.30%	−1.60%	−2.50%	−4.90%	−2.30%	0.90%	1.60%	0.70%
Trochanteric	−2.40%	−5.70%	−3.30%	2.10%	2.80%	0.80%	−4.50%	−8.50%	−4.10%
Intertrochanteric	2.00%	−0.70%	−2.60%	−0.70%	−3.40%	−2.70%	2.70%	2.60%	0.10%
*Average*	−*1.00%*	−*3.10%*	−*2.10%*	*0.50%*	−*0.60%*	−*1.20%*	−*5.30%*	−*6.80%*	−*1.30%*
